# Complete Genome Sequence of Pig-Originated Foot-and-Mouth Disease Virus Serotype O from Bangladesh

**DOI:** 10.1128/genomeA.01150-16

**Published:** 2016-10-27

**Authors:** M. Rahmat Ali, Huzzat Ullah, Mohammad Anwar Siddique, Munawar Sultana, M. Anwar Hossain

**Affiliations:** Department of Microbiology, University of Dhaka, Dhaka, Bangladesh

## Abstract

In this article, we document the first pig-isolated complete genome sequence of foot-and-mouth disease virus type O in Bangladesh. The complete viral genome revealed a potential serotypic recombination at the 5′ untranslated region (UTR). Conventional amino acid deletion was lacking in 3A region, and antigenic heterogeneity to circulatory type O existed within the VP1 region.

## GENOME ANNOUNCEMENT

Foot-and-mouth disease (FMD) is an acute systemic disease of domestic and wild bovids that causes great agroeconomic losses worldwide. The etiological agent, FMD virus (FMDV), is a single-stranded positive-sense RNA virus belonging to the *Aphthovirus* genus of the *Picornaviridae* family (1), which includes seven distinct serotypes (O, A, C, Asia-1, SAT-1, SAT-2, and SAT-3) and multiple subtypes worldwide (2). In Bangladesh, the FMDV type O Ind2001 lineage of the ME-SA topotype was reported to be homogenously distributed across the regions during 2011 and 2012 (3).

Here, we report the complete nucleotide sequence of an FMDV serotype O strain (BAN/GO/Ka-236(Pig)/2015) isolated from vesicular lesion of the feet of an infected pig, collected on 25 August 2015 in Gopalganj, Bangladesh. Viral RNA was extracted from the infected cell culture supernatant at passage 2 in the BHK-21 cell line, and cDNA was synthesized with random and oligo(dT) primers. A total of 16 overlapping amplicons covering the entire viral genome were generated using internal primer pairs, sequenced with an ABI genetic analyzer, and assembled using SeqMan version 7.0 (DNAStar Lasergene, USA). Phylogenetic analysis was performed using the MEGA 5.2 software.

The complete genome of strain BAN/GO/Ka-236(Pig)/2015 is 8,211 nucleotides (nt) in length and contains a 1,099-nt 5′ untranslated region (5′ UTR) with a 16-nt poly(C) tract, a 6,999-nt open reading frame (ORF), and a 113-nt 3′ UTR with a ≥22-nt poly(A) tail. Phylogenetically, the isolated strain clustered within the Ind2001 lineage of the ME-SA topotype of FMDV serotype O, which corroborates with the previous report of the circulatory FMDV type O (3). Computational analysis of the 5′ UTR of the isolate predicted evidence of recombination between 5′ UTR of the circulatory FMDV type A (BAN/GA/Sa-197/2013; accession no. KJ754939) (4) and FMDV type O (BAN/NA/Ha-156/2013; accession no. KF985189) (5).

BAN/GO/Ka-236(Pig)/2015 shares 95% nucleotide homology (in relation to the complete genome) with the previously reported circulatory FMDV type O strain (BAN/NA/Ha-156/2013; accession no. KF985189) (5); however, 3 amino acid substitutions were found in the VP1 region (E110A, T156A, and S197E), indicating antigenic heterogeneity between them. Moreover, despite being an isolate from pig, no amino acid deletion in the encoded 3A protein was evident, which is contradictory to previous literature (1). Also, 41 nucleotide insertions within its 5′ UTR were detected in comparison to the NCBI FMDV type O RefSeq (GenBank accession number NC_004004).

We present here the first whole-genome sequence data for an FMDV type O strain from a naturally infected pig in Bangladesh. This complete genetic information will be helpful for appropriate mapping of circulating strains of FMDV in Bangladesh, including rates of mutations, recombination, and potential transmission routes, which are critical to establish preventive program for this disease. Also, this will help in determining the genetic basis of host specificity of FMDV.

### Accession number(s).

The complete genome sequence of FMDV isolate BAN/GO/Ka-236(Pig)/2015 has been deposited in GenBank database under the accession no. KX712091.
